# Implementing an ICU registry in Ethiopia—Implications for critical care quality improvement

**DOI:** 10.1016/j.jcrc.2024.154525

**Published:** 2024-06

**Authors:** Menbeu Sultan, Ayalew Zewdie, Dilanthi Priyadarshani, Ephrem Hassen, Melkamu Tilahun, Tigist Geremew, Abi Beane, Rashan Haniffa, Sean M. Berenholtz, William Checkley, Bhakti Hansoti, Adam D. Laytin

**Affiliations:** a St. Paul's Hospital Millennium Medical Center, Addis Ababa, Ethiopia; b Addis Ababa Burn, Emergency and Trauma Hospital, Addis Ababa, Ethiopia; c Network for Improving Critical Care Systems and Training, Colombo, Sri Lanka; d Centre for Inflammation Research, University of Edinburgh, Scotland, UK; e Johns Hopkins University School of Medicine, Department of Anesthesia and Critical Care Medicine, Baltimore, MD, USA; f Johns Hopkins University School of Medicine, Department of Internal Medicine, Division of Pulmonary and Critical Care Medicine, Baltimore, MD, USA; g Johns Hopkins University School of Medicine, Department of Emergency Medicine, Baltimore, MD, USA

**Keywords:** Intensive care unit, Critical care, Quality improvement, Outcomes research, Complications, ICU registry, Low- and middle-income countries, Resource-limited setting, Africa

## Abstract

**Purpose:**

Intensive care units (ICUs) in low- and middle-income countries have high mortality rates, and clinical data are needed to guide quality improvement (QI) efforts. This study utilizes data from a validated ICU registry specially developed for resource-limited settings to identify evidence-based QI priorities for ICUs in Ethiopia.

**Materials and methods:**

A retrospective cohort analysis of data from two tertiary referral hospital ICUs in Addis Ababa, Ethiopia from July 2021—June 2022 was conducted to describe casemix, complications and outcomes and identify features associated with ICU mortality.

**Results:**

Among 496 patients, ICU mortality was 35.3%. The most common reasons for ICU admission were respiratory failure (24.0%), major head injury (17.5%) and sepsis/septic shock (13.3%). Complications occurred in 41.0% of patients. ICU mortality was higher among patients with respiratory failure (46.2%), sepsis (66.7%) and vasopressor requirements (70.5%), those admitted from the hospital ward (64.7%), and those experiencing major complications in the ICU (62.3%).

**Conclusions:**

In this study, ICU mortality was high, and complications were common and associated with increased mortality. ICU registries are invaluable tools to understand local casemix and clinical outcomes, especially in resource-limited settings. These findings provide a foundation for QI efforts and a baseline to evaluate their impact.

## Highlights

• ICU mortality is high in Ethiopian ICUs, despite adjusting for illness severity.
• Many ICU deaths are preceded by preventable complications that increase the risk of ICU mortality in this setting.
• ICU registries are powerful tools to guide context-appropriate quality improvement strategies in resource-limited settings.

## 1 Introduction

Low- and middle-income countries (LMICs) are home to 80% of the world's population and have higher burdens of disease and mortality from potentially treatable illnesses than high-income countries (HICs) [1]. Critical care is an essential part of the treatment of the acute life-threatening conditions [2,3]. However, critical care medicine remains in its infancy in many LMICs, with marked disparities between HICs and LMICs in the access to critical care and outcomes of critical illness [4]. The number of ICU beds varies >45-fold between cities in HICs and LMICs [5]. ICU patients with sepsis in LMICs are more than twice as likely to die compared with those in HICs [6].

The Lancet Global Health Commission highlighted the need for high-quality health systems that optimize healthcare in each given context and respond to changing population needs [7]. Efforts to improve quality of critical care in LMICs developed new urgency with the novel challenges posed by the COVID-19 pandemic [8,9]. However, those efforts have been hampered by a paucity of data about critical care capacity, casemix, processes of care and clinical outcomes in those settings [[10], [11], [12]]. ICU registries are clinical databases that compile longitudinal data on casemix, illness severity, clinical outcomes, and increasingly processes of care [[13], [14], [15]]. Data from ICU registries are foundational to develop and evaluate the impact of interventions to improve the outcomes of critically ill patients [16,17]. Although ICU registries were historically considered unfeasible in LMICs, novel cloud-based ICU registry platforms have been successfully implemented in several South Asian countries [[18], [19], [20]]. Data from these LMIC ICU registries have been used to monitor the epidemiology of the critical illnesses, evaluate patient care, identify QI priorities and facilitate participation in international research both prior to and during the COVID-19 pandemic [[21], [22], [23]].

This goal of this study is to demonstrate the utility of a context-appropriate, validated ICU registry that was implemented in the ICUs of two tertiary care facilities in Addis Ababa, Ethiopia to describe the current reality of critical care in that setting including the rates of complications and ICU mortality and identify priority targets for quality improvement efforts.

## 2 Material and methods

This is a retrospective cohort analysis of de-identified ICU registry data that were prospectively collected in two Ethiopian two tertiary referral hospitals, St Paul's Hospital Millennium Medical College (SPHMMC) and Addis Ababa Burn Emergency and Trauma Hospital (AaBET). This analysis includes data for all patients admitted to the adult ICUs at SPHMMC and AaBET over one year from July 1, 2021 to June 30, 2022.

### 2.1 Study setting

Ethiopia is a low-income country in East Africa with a population of 117 million and a per capita gross national income of $960 [24]. Addis Ababa is the nation's capital and largest city, with a population of 5.5 million [25]. Ethiopia has one of the fastest growing economies in the world, increasing an average of 9.5% per year over the past 15 years, with associated reduction in poverty and improvement in human development indicators [24]. Recently, significant efforts have been made to improve the quality and accessibility of acute and critical care services throughout the country under the leadership of the Ethiopian Ministry of Health's Emergency, Injury and Critical Care Directorate [26].

SPHMMC and AaBET have been at the forefront of these efforts. SPHMCC was established as a hospital in 1969, with the addition of a medical school in 2007. It has over 700 inpatient beds and 14 adult ICU beds. It provides a comprehensive array of medical and surgical subspecialty services, including renal replacement therapy, which is not widely available in Ethiopia. SPHMMC established a critical care fellowship in September 2021. AaBET is an affiliate of SPHMMC established in 2015 to improve emergency and critical care, trauma and burn care in the country. With over 350 inpatient beds and 11 adult ICU beds, it is the largest trauma and emergency center in the country.

### 2.2 ICU registry

In June 2021, the ICU registry platform was piloted in the ICUs of SPHMMC and AaBET in collaboration with the United Kingdom Research and Innovation (UKRI) Collaboration for Research Implementation and Training in Africa (CCA) and researchers from the Johns Hopkins University School of Medicine (JHUSOM), an American academic medical center. The structure and function of the registry platform, designed and curated by a Sri Lanka-based not-for-profit organization, has been described previously [18]. The registry was adapted to the local context in Ethiopia in collaboration with the site directors at SPHMMC and AaBET. Adaptations included incorporating local disease patterns, units of measurement for laboratory reporting and processes of care. The registry dataset was assessed for feasibility prior to implementation to mitigate data missingness due to test unavailability. Data collectors were registered nurses with advanced training in critical care who were already working in the ICUs at SPHMMC and AaBET. Data collectors received specialized training on the ICU registry platform and ongoing technical support from the registry implementation team. ICU registry data were compiled daily from clinical records. Data were entered into the ICU registry using tablet computers and uploaded to a secure, cloud-based platform for compilation and storage. SNOMED CT was used to code diagnoses [27]. The study team routinely monitored the ICUs' census in clinical logbooks to ensure that all patients admitted to the ICU were captured in the ICU registry. ICU registry entries were further monitored for missingness of key data elements.

### 2.3 Data analysis

Clinical data were analyzed descriptively using means and standard deviations for normally distributed continuous variables and medians and interquartile ranges for non-normally distributed continuous variables. Analyses addressed the demographics, reason for admission, comorbidities, illness severity, processes of care, medical complications, ICU length of stay and ICU mortality of patients treated in the ICUs at SPHMMC and AaBET. Processes of care included antibiotic administration, vasopressor support, non-invasive and invasive ventilatory support, tracheostomy and renal replacement therapy. Infectious diagnoses such as bacterial pneumonia were made either clinically or with microbiological laboratory data when available. Burden of comorbidities was quantified using the Charlson Comorbidity Index (CCI) [28]. To quantify illness severity and adjust for casemix, SNOMED CT codes were mapped to APACHE II, which ranges from 0 to 71, with increasing score associated with increasing risk of hospital mortality [29], as has been done with other CCA ICU registries [19,20]. Normal imputation was used for missing data. Bivariable and multivariable logistic regression models were used to identify factors associated with hospital mortality, controlling for illness severity. Receiver operating characteristic (ROC) analysis was used to evaluate the ability of APACHE II predicted risk of death (PRoD) to discriminate the outcome of hospital mortality. APACHE II PRoD was used to calculate standardized mortality ratio (SMR), or observed/expected mortality.

### 2.4 Ethical consideration

The study protocol was reviewed and granted approval by the institutional review boards of SPHMMC and JHUSOM. This retrospective analysis of de-identified clinical data was determined to pose minimal risk to patients and the requirement for individual, patient-level informed consent was waived.

## 3 Results

A total of 496 patients were admitted to the ICUs of SPHMMC (*n* = 255) and AaBET (*n* = 241) during the study period, and 175 (35.3%) did not survive the ICU admission. (Table 1) Median age was 35 years (IQR 25–52), and 289 were male 58.3%. The most common comorbidities were hypertension (*n* = 83, 16.7%), type 2 diabetes (*n* = 38, 7.7%) and congestive heart failure (*n* = 31, 6.3%). There were no comorbidities identified in 246 patients (49.6%), with median Charlson Comorbidity Index 0 (IQR 0–2) in both survivors and non-survivors.

**Table 1:** Patient characteristics and illness severity in two Ethiopian ICUs.

Characteristics	All, *n* = 496	Survivors, *n* = 321	Non-Survivors, *n* = 175	*p*-value
**Age in years**, median (IQR)	35 (25–52)	35 (25–51)	35 (26–53)	0.431
**Male,** n (%)	289 (58.3)	194 (60.4)	95 (54.3)	0.218
**Source of admission**, n (%) Emergency department Operating room Ward Transfer from ICU/HDU⁎	230 (46.4) 152 (30.7) 85 (17.1) 29 (5.9)	168 (52.3) 106 (33.0) 30 (9.4) 17 (5.3)	62 (35.4) 46 (26.3) 55 (31.4) 12 (6.9)	<0.001
**Route to admission**, n (%) Non-operative Post-operative⁎⁎ *Emergency surgeries*	303 (61.1) 193 (38.9) *123 (63.7)*	195 (60.7) 126 (39.3) *79 (62.7)*	108 (61.7) 67 (38.3) *44 (65.7)*	0.909 *0.683*
**Most common SNOMED CT disorders reported at admission**, n (%)⁎⁎⁎				
Respiratory failure Major head injury Sepsis/septic shock Aspiration pneumonia Acute renal failure Hospital acquired pneumonia Pulmonary edema Moderate head injury Diffuse axonal brain injury Hemorrhagic shock Other diagnoses	119 (24.0) 87 (17.5) 67 (13.5) 60 (12.1) 39 (7.9) 37 (7.5) 36 (7.3) 31 (6.2) 30 (6.0) 21 (4.2) 154 (31.1)	64 (19.9) 66 (20.6) 22 (6.9) 43 (13.4) 21 (6.5) 21 (6.5) 26 (8.1) 26 (8.1) 25 (7.8) 13 (4.0) 101 (31.5)	55 (31.4) 21 (12.0) 44 (25.1) 17 (9.7) 18 (10.3) 16 (9.1) 10 (5.7) 5 (2.9) 5 (2.9) 8 (4.6) 53 (30.3)	0.006 0.023 <0.001 0.290 0.192 0.382 0.425 0.035 0.045 0.966 0.786
**Most common surgery types at admission in operative patients**, n (%)	*n* = 193	*n* = 126	*n* = 67	
Evacuation of intracranial hematoma Craniotomy Laparotomy Ivor Lewis subtotal esophagectomy Exploration Cesarean section Esophagectomy Splenectomy Appendectomy	43 (22.3) 39 (20.2) 26 (13.5) 6 (3.1) 5 (2.6) 4(0.8) 4(0.8) 4(0.8) 3(0.6)	31 (24.6) 29 (23.0) 18 (14.3) 3 (2.4) 2 (1.6) 1(0.3) 1(0.3) 4(1.2) 3(0.9)	12 (17.9) 10 (14.9) 8 (11.9) 3 (4.5) 3 (4.5) 3(1.7) 3(1.7) 0(0.0) 0(0.0)	0.372 0.255 0.776 0.742 0.489 0.253 0.253 0.338 0.499
**Most common comorbidities reported at ICU admission**, n (%)				
Hypertension Type 2 Diabetes Congestive heart failure Moderate/severe CKD Tuberculosis HIV Cardiovascular disease (excluding hypertension)	83 (16.7) 38 (7.7) 31 (6.3) 26 (5.2) 19 (3.8) 16 (3.2) 16 (3.2)	59 (18.4) 24 (7.5) 18 (5.6) 14 (4.4) 11 (3.4) 10 (3.1) 10 (3.1)	24 (13.7) 14 (8.0) 13 (7.4) 12 (6.9) 8 (4.6) 6 (3.4) 6 (3.4)	0.228 0.974 0.544 0.327 0.697 0.850 0.850
No comorbidities reported at admission, n (%)	246 (49.6)	166 (51.7)	80 (45.7)	0.237
Charlson Comorbidity Index, median (IQR)	0 (0–2)	0 (0–2)	0 (0–2)	0.202
APACHE II score, median (IQR)	15 (11–19)	13 (9–18)	18 (13−22)	<0.001

ICU/HDU intensive care unit/high dependency unit; CKD chronic kidney disease; HIV human immunodeficiency virus.

⁎ ICU/HDU transfers include both patients transferred from ICUs and HDUs within the same hospital and from other institutions.

⁎⁎ Post-operative admissions include both patients transferred directly from the operating room and patients transferred from other parts of the hospital after leaving the operating room.

⁎⁎⁎ More than one SNOMED CT disorder at admission could be reported.

Most patients were admitted to the ICU from the emergency department (*n* = 230, 46.4%) or the operating room (*n* = 152, 30.7%). Of the 193 post-operative patients, 152 were admitted to the ICU from the operating room and the remainder were admitted from other parts of the hospital after leaving the operating room. Most post-operative patients underwent emergency surgery (*n* = 123, 63.7%). The three most common SNOMED diagnoses leading to ICU were respiratory failure (*n* = 119, 24.0%), major head injury (*n* = 87, 17.5%) and sepsis/septic shock (*n* = 66, 13.3%). The three most common SNOMED operative procedures leading to ICU admission among operative patients were evacuation of intracranial hematoma (*n* = 43, 22.3%), craniotomy (for both traumatic and non-traumatic indications) (*n* = 39, 20.2%) and laparotomy (*n* = 26, 13.5%).

**Fig. 1:**
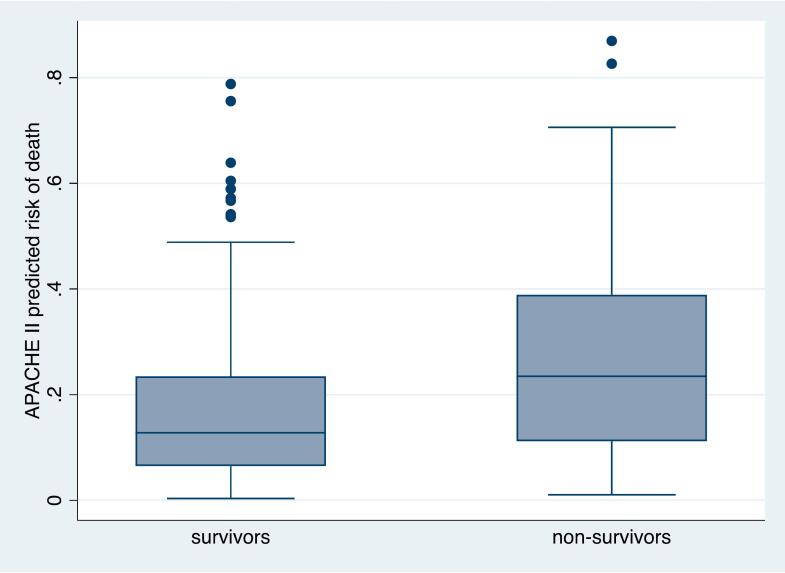
Distribution of APACHE II predicted risk of death among survivors and non-survivors in two Ethiopian ICUs. In these box plots, the horizontal line within the box denotes the median value of the sample, the bottom and top of the box denote the first and third quartiles respectively, the upper whisker extends to the highest value that is within (1.5 x interquartile range) of the third quartile, and the lower whisker extends to the lowest value within (1.5 x interquartile range) of the first quartile. Data beyond the top whisker are outliers and plotted as points.

Each data element necessary to calculate APACHE II score was available in over 90% of patients except for arterial blood pH and arterial partial pressure of oxygen (PaO_2_), which were not measured on any patient in this cohort due to resource limitations. (Supplemental table 1) The median APACHE II score on admission was 15 (IQR 11–19) and the median associated APACHE II PRoD was 0.155 (IQR 0.078–0.293), which was significantly higher among non-survivors than survivors 0.232 (IQR 0.112–0.389) vs 0.128 (IQR 0.066–0.235) (Fig. 1). While APACHE II PRoD correlated well with hospital mortality in a bivariable logistic regression model (*p* < 0.001), the area under the receiver operating characteristic curve was 0.6684 suggesting only moderate discrimination in this mixed ICU population (Supplemental fig. 1). APACHE II PRoD analysis demonstrated an expected mortality rate of 15.5% with SMR 2.28.

Median ICU length of stay was 7 (IQR 3–16) days and was significantly longer among survivors than non-survivors (9 days (IQR 4–20) vs. 4 days (IQR 2–11), p < 0.001). (Table 2) Within 24 h of admission, 362 patients had respiratory failure and received mechanical ventilatory support (73.0%), 105 patients had shock and received vasopressor support (21.2%), and 23 patients had renal failure and received renal replacement therapy (4.6%). During their ICU stays, antibiotics were administered to 403 patients (81.3%) for a median duration of 6 days (IQR 3–12). Mechanical ventilation was administered to 399 patients (80.4%) for a median duration of 6 days (IQR 2–14). Ninety-two patients (18.6%) underwent tracheostomy. Vasopressor support was administered to 152 patients (30.7%). Renal replacement therapy was administered to 34 patients (6.9%).

**Table 2:** Processes of care in the ICU in two Ethiopian ICUs.

	All, n = 496	Survivors, n = 321	Non-Survivors, n = 175	p-value
ICU length of stay in days, median (IQR)	7 (3–16)	9 (4–20)	4 (2−11)	<0.001
**Antibiotics**				
At any time of during ICU stay, n (%)	403 (81.3)	265 (82.6)	138 (78.9)	0.313
Duration in days, median (IQR)	6 (3−12)	7 (3−13)	5 (2−10)	0.002
**Vasopressor support**				
In the first 24 h of ICU admission, n (%)	105 (21.2)	31 (9.7)	74 (42.3)	<0.001
At any time of during ICU stay, n (%)	152 (30.7)	42(13.1)	110(62.9)	<0.001
**Non-invasive ventilatory support**				
In first 24 h of ICU admission, n (%)	6 (1.2)	4 (1.3)	2 (1.1)	0.920
At any time of during ICU stay, n (%)	11 (2.2)	7(2.2)	4(2.3)	0.939
**Invasive ventilatory support**				
In first 24 h of ICU admission, n (%)	362 (73.0)	225 (70.1)	137 (78.3)	0.063
At any time of during ICU stay, n (%)	399 (80.4)	240 (74.8)	159 (90.9)	<0.001
Duration in days, median (IQR)	6 (2–14)	7 (3–16)	4.5 (2–10)	0.014
Tracheostomy during the ICU stay, n (%)	92 (18.6)	70 (21.8)	22 (12.6)	0.011
**Renal replacement therapy**				
In first 24 h of ICU admission, n (%)	23 (4.6)	13 (4.1)	10 (5.7)	0.536
At any time of during ICU stay, n (%)	34 (6.9)	20(6.2)	14(8.0)	0.456

ICU intensive care unit; IQR interquartile range.

Medical complications occurred in 203 patients while in the ICU (41.0%). (Supplemental table 2) The most commonly observed complications were device-related infections—principally catheter-associated urinary tract infections (CAUTIs) and superficial soft tissue infections (SSTIs) around tracheostomy tube and chest tubes (*n* = 78, 15.7%), sepsis (*n* = 70, 14.1%) and bacterial pneumonia (*n* = 38, 7.7%). Cardiac arrest with return of spontaneous circulation (ROSC) occurred in 46 patients (9.3%). Bacteremia and central nervous system infections were very infrequently reported. Major complications (i.e., complications other than device-related infections) occurred in 130 patients (26.2%).

In an unadjusted analysis, ICU mortality was significantly higher among patients transferred to the ICU from the ward than the emergency department or operating room (64.7% vs 27.0% vs 30.2%, *p* < 0.001). ICU mortality was significantly higher among patients requiring vasopressor support within the first 24 h of ICU admission (70.5% vs 25.8%, p < 0.001) and among patients requiring mechanical ventilatory support within the first 24 h of ICU admission (37.9% vs 28.4%, *p* = 0.050). ICU mortality was significantly higher among patients who experienced a major complication (i.e., any medical complication other than a device-related infection) while in the ICU (62.3% vs 25.7%, p < 0.001), with ICU mortality rates of 52.6% among patients who developed bacterial pneumonia in the ICU, 57.1% among patients who developed sepsis in the ICU and 95.7% among patient who suffered cardiac arrest with ROSC. Device-related infections were associated with decreased ICU mortality (14.1% vs 39.2%, p < 0.001).

After adjusting for illness severity using APACHE II PRoD, factors that remained significantly associated with increased ICU mortality included admission from the hospital ward (aOR 5.038, 95% CI 3.000–8.462), history of congestive heart failure (aOR 2.540, 95% CI 1.113–5.719), admission for respiratory failure (aOR 1.747, 95% CI 1.119–2.727), admission for sepsis/septic shock (aOR 3.968, 95% CI 2.239–7.201), need for vasopressor support within 24 h of ICU admission (aOR 5.469, 95% CI 3.339–8.957), and complications including any major complication (aOR 4.992, 95% CI 3.197–7.795), sepsis (aOR 2.989, 95% CI 1.732–5.215), cardiac arrest with ROSC (aOR 62.828, 95% CI 18.406–394.932) and bacterial pneumonia (aOR 2.388, 95% CI 1.176–4.893) (Supplemental table 3). Admission from the emergency department and traumatic brain injury were associated with decreased ICU mortality.

## 4 Discussion

Our analysis found high ICU mortality rates among critically ill patients in this resource-limited setting, with over a third of patients dying prior to ICU discharge. Factors associated with increased ICU mortality in this cohort included patients transferred to the ICU from the hospital ward, those with respiratory failure or sepsis, and those who received vasopressor support. A quarter of patients were diagnosed with at least one medical complication while in the ICU, and adjusted odds of ICU mortality was five times higher among patients who experienced at least one major complication compared with those who did not. While this association does not prove causation, complications such as ICU acquired infections have previously been shown to result in significant increases in ICU LOS and resource utilization, making them important targets for QI efforts [30,31]. In contrast, device-related infections such as CAUTIs and SSTIs were associated with decreased ICU mortality, likely due to immortal time bias.

These findings are consistent with other reports on ICU outcomes in East Africa. Several retrospective cross-sectional studies from adult ICUs in referral medical centers in Ethiopia have reported ICU or hospital mortality rates of 28–50% [[32], [33], [34], [35]], and a recent meta-analysis reported a national ICU mortality rate of 40% in Ethiopia [36]. Because these analyses did not adjust for casemix, direct comparison may be misleading. The associations of increased mortality with respiratory failure, sepsis, receiving for vasopressor support, and infectious and respiratory complications are consistent across these analyses.

Our study is noteworthy among reports on ICU outcomes in Ethiopia both for using prospectively collected data from an ICU registry and for using a critical care prognostic score to adjust for casemix. In our study, SMR suggesting that ICU mortality was 2.28 times higher than would be expected based on illness severity in a resource rich setting. One report on ICU outcomes Kenya that used the Mortality Probability Admission Model II to determine SMR found mortality 2.5 times higher than expected [37]. Studies from South Africa, which is an upper middle income country, using the Simplified Acute Physiology Score 3 to determine SMR found SMR close to 1 [38,39]. Variability in patient populations, resource availability and score selection makes comparison between these studies especially problematic, and SMR has been demonstrated to be a poor indicator of the quality of hospital care [40,41]. In our study, lack of pH and PaO_2_ measurements may result in underestimation of illness severity using APACHE II, however this approach is nevertheless standard practice in this context.

Furthermore, in this analysis APACHE II PRoD had only moderate ability to discriminate ICU mortality. Although APACHE II remains one of the mostly commonly used and researched critical care prognostic scores in LMICs, it has been repeatedly demonstrated to have decreased discrimination and calibration in those settings, which are exacerbated by challenges related to missing data [42,43]. APACHE II has also been shown to underestimate mortality with in patients with traumatic brain injury, which was common in our cohort [44].

The search is ongoing for a simplified critical care prognostic score that is more feasible to implement and more accurate in risk-stratifying a diverse population of patients in resource-limited settings, which is necessary to adjust for casemix in order to accurately evaluate ICU performance [45]. Until a superior prognostic score is identified, APACHE II analysis including SMR does allow for some degree of comparison between healthcare facilities with similar patient populations and resource availability, can be useful for establishing a baseline for evaluating of trends over time and assessing of the impact of QI interventions [46]. Moving forward, developing regional ICU registry networks will allow standardized data collection, prognostic scoring and reporting to allow for more meaningful comparison of ICU outcome data [47].

This study does have several important limitations. Our sample size is relatively small and SPHMMC and AaBET are specialized referral centers; the patterns observed in this analysis may not be generalizable to other ICUs in the region. Due to resource limitations, many diagnoses were made clinically without cross-sectional imaging or microbiological culture data for confirmation. This may result in the underdiagnosis of some conditions. For example, common ICU complications such as pulmonary embolism and acute respiratory distress syndrome were notably infrequent in this cohort, which may result from misclassification as pneumonia. Nevertheless, we believe that this report is useful in demonstrating the importance of high-quality clinical data from an ICU registry to inform QI efforts.

Critical care capacity building is essential in LMICs [7]. The COVID-19 pandemic highlighted the life-saving potential for context-appropriate critical care services targeting the support of acutely failing organ systems [48]. The landmark African COVID-19 Critical Care Outcomes Study reported a 48% hospital mortality rate in patients admitted to ICUs in ten African countries with COVID-19 in 2020, with estimated excess mortality of 11–23 deaths per 100 patients compared with the global average [49].

Efforts to improve the quality of critical care services in resource-limited settings rely on accurate measurements of casemix, processes of care, complications and clinical outcomes [50]. The results of this analysis are useful in identifying several focus areas for quality improvement in our context, including developing and implementing protocols to prevent common medical complications in the ICU, instituting a rapid response team to begin resuscitation and stabilization of critically ill patients on the hospital ward, improving monitoring of patients with shock physiology and ventilator-dependent respiratory failure, and expanding training for ICU providers with particular focus on ventilator management, vasopressor use and sepsis. Several of these efforts are ongoing, bolstered by the establishment of a critical care fellowship training program at SPHMMC in 2022.

## Author statement

All authors have met participatory requirements for authorship. Details are provided in the manuscript submission portal. We have no conflicts of interest. Grant funding is acknowledged in financial disclosures.

## CRediT authorship contribution statement

**Menbeu Sultan:** Conceptualization, Data curation, Formal analysis, Investigation, Methodology, Project administration, Resources, Supervision, Validation, Writing – review & editing. **Ayalew Zewdie:** Conceptualization, Data curation, Formal analysis, Investigation, Methodology, Project administration, Resources, Supervision, Validation, Writing – review & editing. **Dilanthi Priyadarshani:** Formal analysis, Methodology, Writing – review & editing. **Ephrem Hassen:** Data curation, Project administration, Writing – review & editing. **Melkamu Tilahun:** Data curation, Project administration, Writing – review & editing. **Tigist Geremew:** Data curation, Project administration, Writing – review & editing. **Abi Beane:** Conceptualization, Formal analysis, Funding acquisition, Methodology, Resources, Software, Writing – review & editing. **Rashan Haniffa:** Conceptualization, Formal analysis, Funding acquisition, Methodology, Resources, Software, Writing – review & editing. **Sean M. Berenholtz:** Conceptualization, Formal analysis, Methodology, Writing – review & editing. **William Checkley:** Conceptualization, Formal analysis, Methodology, Writing – review & editing. **Bhakti Hansoti:** Conceptualization, Formal analysis, Methodology, Writing – review & editing. **Adam D. Laytin:** Conceptualization, Formal analysis, Funding acquisition, Investigation, Methodology, Resources, Writing – original draft.

## Declaration of competing interest

None.

## Appendix A Supplementary data

Supplementary Figure 1. Receiver operating characteristic curve Image 1

Supplementary table 1. Availability of key data elements Image 2

Supplementary table 2. ICU complications NOTE--the yellow highlighting should be removed from this table for publication Image 3

Supplementary table 3. Factors associated with ICU mortality in adjusted models Image 4
