# Supplementary material for: Implementing an ICU registry in Ethiopia—Implications for critical care quality improvement
Source: J Crit Care. 2024 Jun;81:None. doi: 10.1016/j.jcrc.2024.154525 (PMC10996997; doi:10.1016/j.jcrc.2024.154525)
Supplement: Supplementary file 1 — Supplementary Figure 1. Receiver operating characteristic curve [file mmc1.docx]

Supplemental figure 1: Receiver operating characteristic curve demonstrating association between APACHE II predicted risk of death and ICU mortality in two Ethiopian ICUs
