# Supplementary material for: Implementing an ICU registry in Ethiopia—Implications for critical care quality improvement
Source: J Crit Care. 2024 Jun;81:None. doi: 10.1016/j.jcrc.2024.154525 (PMC10996997; doi:10.1016/j.jcrc.2024.154525)
Supplement: Supplementary file 2 — Supplementary table 1. Availability of key data elements [file mmc2.docx]

Supplementary Table 1: Availability of key data elements in two Ethiopian ICUs

| **Variable** | **Availability, n (%)** |
| --- | --- |
| Age | 496 (100.0) |
| Temperature | 495 (99.8) |
| Mean arterial pressure | 495 (99.8) |
| Heart rate | 495 (99.8) |
| Respiratory rate | 495 (99.8) |
| GCS | 495 (99.8) |
| FiO_2_ | 460 (92.7) |
| Serum pH | 0 (0.0) |
| PaO_2_ | 0 (0.0) |
| Serum sodium | 454 (91.5) |
| Serum potassium | 453 (91.3) |
| Serum creatinine | 466 (94.0) |
| Serum bicarbonate | 0 (0.0) |
| Hematocrit | 489 (98.6) |
| White blood cell count | 484 (97.6) |
| Acute renal failure | 496 (100.0) |

GCS Glasgow coma score; FiO_2_ fraction of inspired oxygen; PaO_2_ partial pressure of arterial oxygen

Each data element listed is necessary to calculate APACHE II score, except for serum bicarbonate. All data elements necessary to calculate APACHE II score were available in over 90% of patients except for arterial blood pH and PaO_2_, which was not measured on any patient in this cohort due to resource limitations.
