# Supplementary material for: Implementing an ICU registry in Ethiopia—Implications for critical care quality improvement
Source: J Crit Care. 2024 Jun;81:None. doi: 10.1016/j.jcrc.2024.154525 (PMC10996997; doi:10.1016/j.jcrc.2024.154525)
Supplement: Supplementary file 3 — Supplementary table 2. ICU complications NOTE--the yellow highlighting should be removed from this table for publication [file mmc3.docx]

Supplemental table 2: ICU complications in two Ethiopian ICUs

|  | All,  n = 496 | Survivors,  n = 321 | Non-Survivors,  n = 175 | p-value |
| --- | --- | --- | --- | --- |
| Any complication reported during the ICU stay, n (%) | 203 (41.0) | 113 (35.2) | 90 (51.4) | <0.001 |
| **Complications during ICU stay, n (%)** |  |  |  |  |
| Device-related infection | 78 (15.7) | 67 (20.9) | 11 (6.3) | <0.001 |
| Major complications* | 130 (26.2) | 49 (15.3) | 81 (46.3) | <0.001 |
| Sepsis (not identified at admission) | 70 (14.1) | 30 (9.4) | 40 (22.9) | <0.001 |
| Cardiac arrest with ROSC | 46 (9.3) | 2 (0.6) | 44 (25.1) | <0.001 |
| Bacterial pneumonia | 38 (7.7) | 18 (5.6) | 20 (11.4) | 0.020 |
| Pneumothorax | 8 (1.6) | 5 (1.6) | 3 (1.7) | 0.580 |
| Pulmonary embolism | 6 (1.2) | 1 (0.3) | 5 (2.9) | 0.022 |
| Arrhythmia | 4 (0.8) | 3 (0.9) | 1 (0.6) | 0.558 |
| Glycemic emergency | 2 (0.4) | 0 (0.0) | 2 (1.1) | 0.124 |
| ARDS | 1 (0.2) | 0 (0.0) | 1 (0.6) | 0.353 |
| CNS infection | 1 (0.2) | 0 (0.0) | 1 (0.6) | 0.353 |
| DIC | 1 (0.2) | 0 (0.0) | 1 (0.6) | 0.353 |
| Pancreatitis | 1 (0.2) | 0 (0.0) | 1 (0.6) | 0.353 |
| Anemia | 1 (0.2) | 1 (0.3) | 0 (0.0) | 0.647 |
| Bacteremia | 1 (0.2) | 1 (0.3) | 0 (0.0) | 0.647 |
| Stroke | 1 (0.2) | 1 (0.3) | 0 (0.0) | 0.647 |

ICU intensive care unit; ROSC return of spontaneous circulation; ARDS acute respiratory distress syndrome; CNS central nervous system; DIC disseminated intravascular coagulation

*Major complications refer to all complications other than device-related infections

This table presents unadjusted comparisons of incidence of complications between survivors and non-survivors in the ICU.
