# Supplementary material for: Implementing an ICU registry in Ethiopia—Implications for critical care quality improvement
Source: J Crit Care. 2024 Jun;81:None. doi: 10.1016/j.jcrc.2024.154525 (PMC10996997; doi:10.1016/j.jcrc.2024.154525)
Supplement: Supplementary file 4 — Supplementary table 3. Factors associated with ICU mortality in adjusted models [file mmc4.docx]

Supplemental table 3: Factors associated with ICU mortality in adjusted models controlling for APACHE II predicted risk of death in two Ethiopian ICUs

| Factor | aOR | 95% CI | p-value |
| --- | --- | --- | --- |
| **Age** (years) | 0.999 | 0.989-1.010 | 0.920 |
| **Male** | 0.687 | 0.465-1.015 | 0.060 |
| **Source of admission** |  |  |  |
| Emergency Department | 0.555 | 0.374-0.822 | 0.003 |
| Operating theater | 1.301 | 0.805-2.098 | 0.280 |
| Ward | 5.038 | 3.000-8.462 | <0.001 |
| Transfer from ICU/HDU | 1.392 | 0.629-3.085 | 0.416 |
| **Route to admission** |  |  |  |
| Post-operative (vs non-operative) | 0.735 | 0.489-1.105 | 0.139 |
| emergency surgical (vs elective surgical) | 0.750 | 0.378-1.487 | 0.410 |
| **SNOMED CT primary disorders reported at admission** |  |  |  |
| Respiratory failure  Major head injury  Sepsis/septic shock  Aspiration pneumonia  Acute renal failure  Hospital acquired pneumonia  Pulmonary edema  Moderate head injury  Diffuse axonal brain injury  Hemorrhagic shock | 1.747  0.409  3.968  0.597  1.313  1.174  0.759  0.314  0.316  0.982 | 1.119-2.727  0.232-0.698  2.239-7.201  0.313-1.092  0.649-2.628  0.563-2.407  0.323-1.660  0.103-0.786  0.104-0.786  0.362-2.510 | 0.013  0.001  <0.001  0.104  0.442  0.661  0.505  0.022  0.022  0.972 |
| **Surgery types at admission in operative patients** |  |  |  |
| Evacuation of intracranial hematoma  Craniotomy  Laparotomy  Ivor Lewis subtotal esophagectomy  Exploration  Cesarean section  Esophagectomy | 0.483  0.492  1.123  1.741  3.529  6.332  5.373 | 0.225-0.972  0.218-1.027  0.434-2.687  0.301-10.021  0.546-29.000  0.729-135.930  0.615-114.702 | 0.049  0.070  0.800  0.516  0.188  0.126  0.163 |
| **Comorbidities**  Presence of any comorbidity  Hypertension  Type 2 Diabetes  Congestive heart failure  Moderate/severe CKD  Tuberculosis  HIV  Cardiovascular disease (excluding hypertension) | 1.113  0.656  0.860  2.540  0.727  2.204  1.484  1.404 | 0.817-1.695  0.373-1.122  0.398-1.784  1.113-5.719  0.301-1.715  0.798-5.895  0.465-4.380  0.443-4.129 | 0.322  0.132  0.692  0.024  0.469  0.116  0.482  0.543 |
| **Processes of care in the first 24 hours of ICU admission** |  |  |  |
| Vasopressor support | 5.469 | 3.339-8.957 | <0.001 |
| Invasive ventilatory support | 0.994 | 0.626-1.579 | 0.981 |
| Renal replacement therapy | 1.306 | 0.538-3.168 | 0.555 |
| **Complications during ICU stay** |  |  |  |
| Device-related infection | 0.193 | 0.092-0.372 | <0.001 |
| Major complications  Sepsis | 4.992  2.989 | 3.197-7.795  1.732-5.215 | <0.001  <0.001 |
| Cardiac arrest | 62.828 | 18.406-394.932 | <0.001 |
| Bacterial pneumonia | 2.388 | 1.176-4.893 | 0.016 |

aOR adjusted odds ratio; ICU intensive care unit; HDU high dependency unit

Major complications include any medical complication other than a device-related infection in this sample
